# A Perspective on Nrf2 Signaling Pathway for Neuroinflammation: A Potential Therapeutic Target in Alzheimer's and Parkinson's Diseases

**DOI:** 10.3389/fncel.2021.787258

**Published:** 2022-01-21

**Authors:** Sarmistha Saha, Brigitta Buttari, Elisabetta Profumo, Paolo Tucci, Luciano Saso

**Affiliations:** ^1^Department of Cardiovascular, Endocrine-Metabolic Diseases and Aging, Italian National Institute of Health, Rome, Italy; ^2^Department of Clinical and Experimental Medicine, University of Foggia, Foggia, Italy; ^3^Department of Physiology and Pharmacology “Vittorio Erspamer”, Sapienza University of Rome, Rome, Italy

**Keywords:** Alzheimer's disease, Parkinson's disease, Nrf2 signaling pathway, neuroinflammation, oxidative stress, Keap1

## Abstract

Neuroinflammation plays a pivotal role in Alzheimer's disease (AD) and Parkinson's disease (PD), the leading causes of dementia. These neurological disorders are characterized by the accumulation of misfolded proteins such as amyloid-ß (Aß), tau protein and α-synuclein, contributing to mitochondrial fragmentation, oxidative stress, and neuroinflammation. Misfolded proteins activate microglia, which induces neuroinflammation, expression of pro-inflammatory cytokines and subsequently facilitates synaptic damage and neuronal loss. So far, all the proposed drugs were based on the inhibition of protein aggregation and were failed in clinical trials. Therefore, the treatment options of dementia are still a challenging issue. Thus, it is worthwhile to study alternative therapeutic strategies. In this context, there is increasing data on the pivotal role of transcription factor NF- E2 p45-related factor 2 (Nrf2) on the redox homeostasis and anti-inflammatory functions in neurodegenerative disorders. Interestingly, Nrf2 signaling pathway has shown upregulation of antioxidant genes, inhibition of microglia-mediated inflammation, and improved mitochondrial function in neurodegenerative diseases, suggesting Nrf2 activation could be a novel therapeutic approach to target pathogenesis. The present review will examine the correlation between Nrf2 signaling with neuroinflammation in AD and PD.

## Introduction

Neuroinflammation is a crucial hallmark in the progression of neurodegenerative conditions such as Alzheimer's disease (AD), Parkinson's disease (PD), Huntington's disease, multiple sclerosis, Friedrich's ataxia, and stroke (Stephenson et al., 2018). Alzheimer's disease (AD), the most common neurological disorder is an irreversible progressive neurodegenerative disease characterized by abnormal aggregation of amyloid β-peptide (Aβ), and hyperphosphorylated tau protein (p-tau) accumulation leading to the neuroinflammation, oxidative stress and a gradual loss in cholinergic, synaptic and cognitive functions (Li and Götz, 2017). Parkinson's disease (PD), the second most common neurological disorder, is characterized by progressive degeneration and death of dopaminergic neurons and the characteristic feature is the formation of fibrillar aggregates into intraneuronal inclusions, called Lewy bodies (LBs) which constitute more than 70% of a-synuclein (Mahul-Mellier et al., 2020). Protein misfolding, mitochondrial damages, oxidative stress and inflammation are the primary risk factors in AD and PD.

A common feature of all neurodegenerative diseases is immense oxidative stress leading to the dysfunction of neuronal cells. Oxidative stress is a biological condition driven by the imbalance between reactive oxygen species (ROS) production and cellular antioxidant defense response. Oxidative stress cause membrane lipid oxidation, ROS attack cellular membranes leading to functional and/or structural impairment of the membranes and to the formation of toxic lipid products as 4-hydroxy-2,3-nonenal (HNE), malondialdehyde, acrolein, and F2-isoprostanes. In respect of their oxidative-induced damage properties, these compounds are considered as disease mediators and due to their more stable forms their measure render quantifiable the magnitude of oxidative stress in biological samples (Erejuwa et al., 2013; Sultana et al., 2013). Indeed, the brain tissue in AD and PD and the cerebrospinal fluid (CSF) of ALS patients showed high levels of HNE (Dexter et al., 1989; Pedersen et al., 1998; Selley et al., 2002). Similarly, thiobarbituric acid-reactive substances (TBARs), acrolein, and F2-isoprostanes are all found to be elevated in AD (Arlt et al., 2002) and PD brains (Dexter et al., 1989), whereas elevated TBARs have been observed in the plasma of amyotrophic lateral sclerosis (ALS) patients (Sayre et al., 2001). As an endogenous defense mechanism, the activity of the antioxidant proteins such as catalase, superoxide dismutase (SOD), glutathione peroxidase and glutathione reductase are significantly up-regulated in the hippocampus and amygdala of AD brains (Pappolla et al., 1992). Furthermore, Aβ42 binds copper (I) ions forming Aβ42-Cu^+^ complex which could reduce oxygen to generate H_2_O_2_ and free radicals (Jiang et al., 2007).

It is well established that the development and progression of PD involved oxidative stress, mitochondrial dysfunction, and also neuroinflammation (Di Filippo et al., 2010). *In vivo* and *in vitro* studies have shown disruption of mitochondrial function in the dopamine neurons in the substantia nigra in early stages of PD (Hattingen et al., 2009), and decreased enzymes activity in the electron transport chain has been observed throughout the course of the disease (Schapira et al., 1989, 1990; Trimmer et al., 2000; Tysnes and Storstein, 2017). Moreover, increased mutations in mitochondrial DNA (mtDNA) with impaired Complex I and consequently increased oxidative stress was observed in later stages of PD patients, (Schapira, 2008; Moon and Paek, 2015).

In PD, α-synuclein has a mitochondrial targeted amino-terminal sequence which is responsible for the interactions with the inner mitochondrial membrane and disruption of complex I function, thereby triggers oxidative stress (Chinta et al., 2010). Moreover, the parkin protein, which is constitutively expressed in normal mitochondria, reportedly found to be inhibited with impaired complex I activity in oxidative stress conditions (Muftuoglu et al., 2004). In addition, DJ-1, a protein with antioxidant properties, is a well-known oxidative stress sensor as excess oxidation of DJ-1, renders this protein inactive. The oxidized form of DJ-1 protein has been observed in patients with sporadic PD and AD, suggesting the role of DJ-1 in the onset and pathogenesis of sporadic PD as well as familial PD (Cookson, 2010). In response to oxidative stress, nuclear factor (erythroid-derived 2)-like2 (Nrf2) plays a crucial role by inducing expression of a wide range of cytoprotective genes. Overexpression of DJ-1 has been reported to increase the Nrf2 protein levels and enhances its antioxidant role to improve the phase II response (Im et al., 2012).

Nrf2 plays a crucial role in regulation of cellular redox homeostasis and neuroinflammation. Accumulating evidences indicated the expression of Nrf2 in neurons, astrocytes, and glial cells (Cuadrado et al., 2019). Interestingly, Nrf2 expression is found to be higher in astrocytes than in neurons and activation of Nrf2 triggers Nrf2 target genes in astrocytes (Lee et al., 2003). Furthermore, astrocytes overexpressing Nrf2 protects neurons from oxidative stress (Johnson et al., 2008).

## Nrf2-Keap1 Signaling Pathway

**Figure 1:**
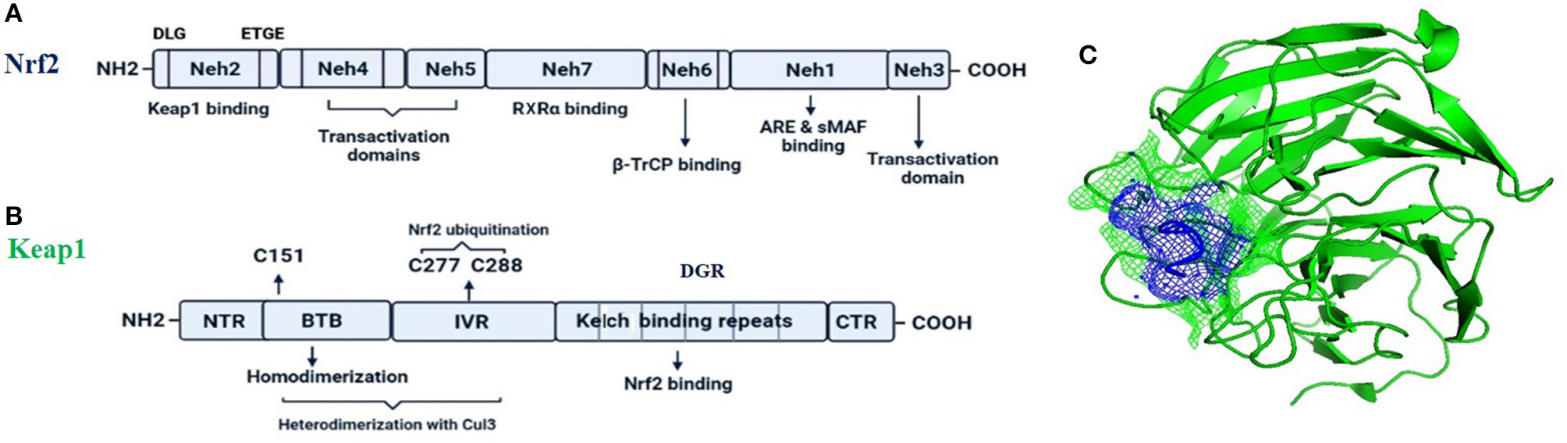
The fundamental structures of Nrf2 **(A)** and Keap1 **(B)**. In **(C)** is reported a surface presentation of the N-terminal region of the Nrf2 (purple color in mesh) in complexed with the Keap1 protein (green color in carton) from crystal structures: 2DYH.

Nrf2 (NF-E2-related factor 2), is a member of the Cap'n'collar (CNC) transcription factor family and involved in redox signaling, xenobiotic metabolism (Lin et al., 2015), metabolism of carbohydrates (Heiss et al., 2013), lipids and iron (Chambel et al., 2015), antioxidant responses, and anti-inflammatory responses. Nrf2 protein consists of 605 amino acids and is divided into seven highly conserved functional domains, namely Neh1-Neh7 (Figure 1A). The Neh1 domain has a cap “n” collar basic-region leucine zipper (bZIP) domain, which is responsible for DNA-binding (Sun et al., 2009) and a nuclear localization signal (NLS) that regulates nuclear translocation of Nrf2 (Theodore et al., 2008). The Neh3, Neh4, and Neh5 are transactivation domains which regulates the binding of Nrf2 with other coactivators (Nioi et al., 2005). The Neh6 domain acts as a negative regulatory domain and binds with a β-transducin repeat-containing protein (β-TrCP) for Nrf2 ubiquitination (Rada et al., 2012). The Neh7 domain is involved in the direct binding to the retinoic X receptor α (RXRα), a repressor of NRF2, thus contributing to the inhibition of Nrf2-ARE signaling pathway (Wang et al., 2013). On the other hand, the Neh2 domain constitutes an N-terminal regulatory domain and regulates the stability of Nrf2 by influencing binding with different proteins. Neh2 domain consists of seven lysine residues which are responsible for ubiquitin conjugation. In addition, it also consists of two peptide-binding motifs (DLG and ETGE), which interact with Keap1 and is responsible for Nrf2 ubiquitination and its proteasomal degradation under normal physiological conditions (Lin et al., 2015).

Keap1, the main intracellular regulator of Nrf2, is a cysteine-rich protein, which is divided into five domains (Figure 1B), an N-terminal region (NTR), a Tramtrack-Bric-a-Brac (BTB) domain, a central intervening region (IVR) with a nuclear export signal (NES) regulating the cytoplasmic localization of Keap1, six Kelch repeats, and a C-terminal domain (CTR) (Ogura et al., 2010).

Under normal conditions, Nrf2 is sequestered by cytoplasmic Keap1 and targeted to proteasomal degradation (Wakabayashi et al., 2003). During homeostasis, the BTB domain regulates Keap1 homodimerization and its binding to the cullin-based (Cul3) E3 ligase, forming Keap1-Cul3-RBX1 (Ring box protein-1) (Figure 1C) E3 ligase complex (Zipper and Mulcahy, 2002), whereas Kelch repeats are reportedly regulate the binding of Keap1 to Nrf2 and p62 (Hayes and McMahon, 2009; Komatsu et al., 2010).

**Figure 2:**
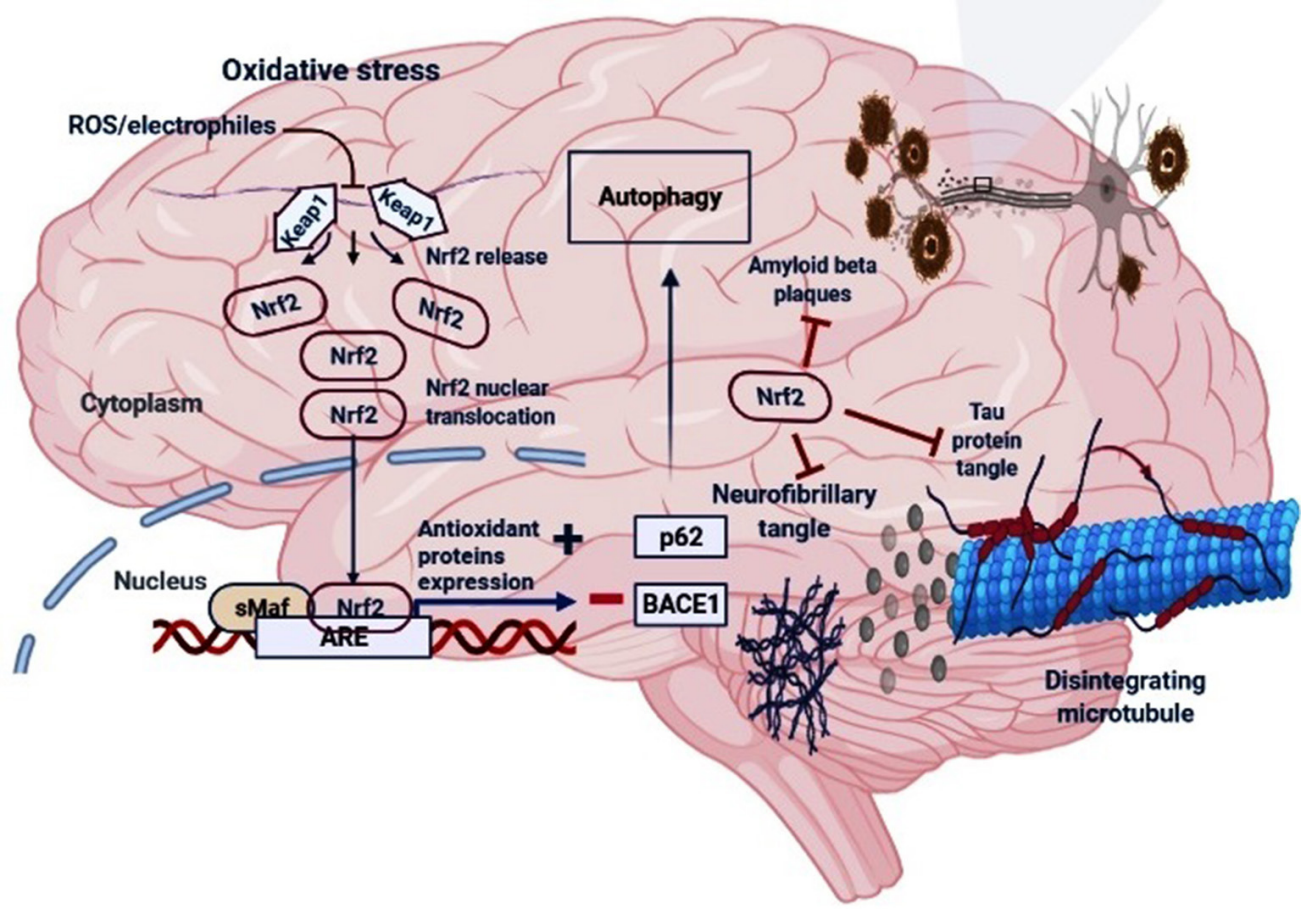
The regulatory mechanism of Nrf2 in AD. Under oxidative stress (electrophiles or ROS conditions) Nrf2 is released from Keap1-Cul3-RBX1 complex for translocation into the nucleus followed by its heterodimerization with sMaf which leads to its binding with the antioxidant response elements (AREs), and transcription of ARE-driven genes. Nrf2 activation may increase the levels of p62 which is responsible for the autophagic process and inhibit the BACE1 that generate amyloid-β peptides in the neurons. Moreover, Nrf2 counteracts neurofibrillary, tau proteins tangle and amyloid-β plaques.

During oxidative stress conditions, the DLG motif dissociates from Keap1 protein leading to a disruption in the alignment of Nrf2 lysine residues that prevents its ubiquitination and consequently, Nrf2 is released from Keap1-Cul3-RBX1 complex, translocate into the nucleus and heterodimerizes with one of the sMaf (musculoaponeurotic fibrosarcoma oncogene homolog) proteins (Suzuki and Yamamoto, 2015) and up-regulates electrophile response element (EpRE)-mediated transcription (Itoh et al., 1999). This activates the transcription of a cascade of genes containing an antioxidant response element (ARE) within their promoter region (Hayes et al., 2010). However, the binding of DGR domain to Nrf2 is competitively inhibited by proteins with specific motifs, such as p62 and localizer of BRCA2 (Keum and Choi, 2014; Canning et al., 2015; Lu et al., 2016), thus responsible for sensing of cellular stress (Rachakonda et al., 2008). A non-canonical pathway for activation of Nrf2 involves competitive inhibition of the Keap1-Nrf2 interaction *via* p62/Sqstm1 (Komatsu et al., 2010; Figure 2). In case of p62-Keap1-Nrf2 axis, p62 acts as a modulator of Nrf2 activation. The Keap1-interacting region (KIR) of p62 interacts with Keap1, preventing Keap1 from trapping Nrf2, which leads to the Nrf2 stabilization following its activation (Komatsu et al., 2010). The KIR region of p62 consists of serine 349, which is phosphorylated during oxidative stress conditions. The phosphorylated p62 in turn has a higher affinity for Keap1 (Ichimura et al., 2013), and is responsible for the interference of Keap1-mediated Nrf2 ubiquitination.

Another model, Nrf2-EpRE pathway regulation *via* nicotinic receptors postulates that in oxidative stress conditions, after receptor activation, post-translational modifications occurs in Nrf2 which stimulates nuclear translocation and binding of Nrf2 with the EpRE sequences (Parada et al., 2014). Interestingly, this model represents a correlation between anti-inflammatory pathway and the Nrf2-dependent phase II antioxidant regulation (Martelli et al., 2014). The phosphorylation of Nrf2 by different kinases has been reported to affect the Nrf2 translocation. Phosphorylation of Ser40 residue of Nrf2 by atypical PKC iota (aPKCι) releases Nrf2 from Keap1 (Bloom and Jaiswal, 2003), allowing Nrf2 transport to the nucleus (Bloom and Jaiswal, 2003; Numazawa et al., 2003). Similarly, other kinases such as casein kinase-2 (CK2) (Pi et al., 2007), c-Jun N-terminal kinase (JNK) and extracellular regulated kinase (ERK) (Keum et al., 2006), and phosphatidylinositide-3-kinases (PI3K) (Nakaso et al., 2003) are also involved in the activation of Nrf2 translocation to the nucleus.

Several kinases are constitutively activated or over-expressed in chronic inflammation or oxidative stress conditions. Glycogen synthase kinase 3-beta (GSK3β) can phosphorylate Nrf2 leading to the recognition of Nrf2 by an E3 ligase receptor and the F-box protein β-TrCP followed by its degradation in a Keap1-independent manner (Chowdhry et al., 2013). It has been shown that the Neh6 domain of Nrf2 consists of two motifs, the activity of one of which, DSGIS, is significantly up-regulated by GSK3β activity (Chowdhry et al., 2013). On the other hand, accumulating evidences also indicated that, GSK3β activation could phosphorylate Fyn, which in turn regulates Nrf2 *via* phosphorylation, nuclear export and proteasomal degradation during pathological conditions (Jain and Jaiswal, 2007). Another kinase, MAPK p38 reportedly stabilizes the interaction between Keap1 and Nrf2 thereby induces the Nrf2 breakdown (Keum et al., 2006).

## Role of Nrf2 in Alzheimer's Disease

AD affects more than 50 million people, and medical management is still a challenge as its pathogenesis still needs to be explored (Zhang et al., 2021). The pathogenesis in AD is related to the aggregation of Aß plaques and hyperphosphorylation of the microtubule-associated protein, tau resulting in neurofibrillary tangles (NFTs). The AßPP processing by proteases leads to the generation of Aß, which is then transferred from the brain to the cerebrospinal fluid (CSF) and is engulfed by microglia by phagocytosis (Heneka et al., 2015). On the other hand, hyperphosphorylated tau protein forms oligomeric paired helical fragments (PHFs), leading to intracellular NFTs, forming abnormal aggregates.

The level of Nrf2 is reportedly decrease as a function of age (Zhang et al., 2015) and observed to be reduced in AD patients (Ramsey et al., 2007). Accumulating evidences also suggested a significant negative correlation between Nrf2 deficits and AD (Zhang et al., 2015; Rojo et al., 2017), which might be due to the fact that the transcription factor, Nrf2 is responsible for the amelioration of oxidative stress and inflammation. Also, Nrf2 directly and indirectly influences changes in autophagy *in vivo* and *in vitro* (Riley et al., 2014; Joshi et al., 2015). Some other reports have shown the cognitive deficits in AD animal models and aggravates AD-like pathology *via* Nrf2 ablation (Joshi et al., 2015; Rojo et al., 2017). Furthermore, activation of Nrf2 by genetic and pharmaceutical inteventions leads to a neuroprotective role in AD patients (Bahn and Jo, 2019).

Nrf2 target genes such as NADPH quinone oxidoreductase I (NQO1), Heme oxygenase-1 (HO-1), and glutamate-cysteine ligase catalytic subunit (GCLC) expressions were observed in AD brains (Silva-Palacios et al., 2018). Nrf2-regulated target genes, NQO1 and NQO2, are two cytosolic flavoproteins responsible for the catalysis of two-electron mediated reduction of quinones to hydroquinones (Ross and Siegel, 2017). NQO1 maintains the reduced form of CoQ9 and CoQ10 inside the lipid vesicles and thus protects the plasma membrane from membrane lipid peroxidation and free radicals. A number of evidence suggest a NQO1 role in development and progression of AD (Chhetri et al., 2018). In this respect, it was reported that NQO1 enzyme activity is up-regulated in the brain areas involved in AD pathology such as frontal cortex (SantaCruz et al., 2004). Another recent study showed the elevation in NQO1 expression in 3xTg-AD mice preceded any intraneuronal Aβ immunoreactivity suggesting that up-regulation of NQO1 in AD pathology (Bahn et al., 2019). In contrast, in another study performed in AD human and mouse models, it has been demonstrated that Nrf2 activation was not able to regulate some of its target genes thus determining repressed expression of antioxidant defenses (Mota et al., 2015). This discordance could depend on the stage of disease. In fact, during the initial phases of AD, Nrf2-dependent gene expression is up-regulated due to the initial defensive cellular mechanism against ROS, however in the latter stages, as oxidative stress increases, Nrf2-dependent gene expression was shown to either reduced or remains stationary (Ansari and Scheff, 2010). The protective role of Nrf2 in AD is supported by results derived from Nrf2-deficient mice. In fact, Nrf2^−/−^ mice crossed with mutant APP/PS1 mice leads to the increase in Aβ and AβPP intracellular levels compared to mutant AβPP/PS1 mice (Joshi et al., 2015). A significant increase was also observed in the insoluble p-tau and Aβ levels in Nrf2-deficient mice was observed (Rojo et al., 2017). It was shown that the lack of Nrf2 significantly worsens cognitive deficits in the APP/PS1 mouse model of AD (Branca et al., 2017).

Impaired proteostasis is a crucial hallmark of neurodegenerative diseases (Hara et al., 2006; Inoue et al., 2012). Macroautophagy is one of the main mechanisms that ensure timely degradation of misfolded, oxidized or altered proteins that otherwise develop proteinopathy (Ciechanover and Kwon, 2015). A functional connection between Nrf2 and macroautophagy gene expression was shown in a mouse model of AD that reproduces impaired APP (amyloid β precursor protein) and human (Hs)MAPT/Tau processing, clearance and aggregation (Pajares et al., 2016). Nrf2-regulated autophagy marker SQSTM1/p62 was observed to be reduced in the absence of Nrf2 (Pajares et al., 2016). Other reports stated that Nrf2 has an impact on chaperone-mediated autophagy (Pajares et al., 2018). Nrf2 binding sequences were identified in the LAMP2 (lysosomal associated membrane protein 2A) gene in several human and mouse cell types and Nrf2 deficiency and overexpression was found to be correlated with reduced and increased LAMP2A levels, respectively (Pajares et al., 2018).

Nrf2 deletion in APP/PS1 mice reportedly enhanced inflammatory response and increase in intracellular APP, Aβ42 and Aβ40 levels. Mechanistically, neurons from Nrf2-deficient APP/PS1 mice shows enhanced accumulation of endosomes, lysosomes, and multivesicular bodies (Joshi et al., 2015). These findings indicated Nrf2-dependent processing and accumulation of APP/Aβ, and autophagic dysfunction. In agreement with these findings, *in vivo* Nrf2 activation in response to the AD-initiating Aβ42 peptide, was shown to prevent neuronal toxicity (Kerr et al., 2017). Another study in the AD animal model reported the reduction in Aβ42 level and p-tau after treatment with Nrf2 activator, isoastilbin (Yu et al., 2019). Accordingly, further analysis revealed that Nrf2 inhibits the beta-site amyloid precursor protein cleaving enzyme 1 (BACE1) expression by binding to the AREs in the promoter of BACE1 in AD animal models. This further inhibits Aβ production, and ameliorates cognitive deficits, however, Nrf2-dependent regulation of BACE1 is independent of ROS repression (Bahn et al., 2019). BACE1 is a beta secretase that generate amyloid-β peptides in the neurons.

Importantly, Nrf2 could reduce the levels of p-tau in AD by inducing nuclear dot protein 52 (NDP52) by binding to the AREs in the promoter of NDP52 (Jo et al., 2014). NDP52, is an autophagy-associated protein which facilitates autophagy-mediated degradation of p-tau (Jo et al., 2014). Consistent with this, another study reported the Nrf2-dependent modulation of selective autophagy processes which facilitates the clearance of tau species (Tang et al., 2018). The down-regulation in the expression of BAG3, NBR1, NDP52, and p62 genes were observed in aged Nrf2^−/−^ animals compared to those in young Nrf2^−/−^ animals, suggesting the role of Nrf2 in gene expressions during aging (Tang et al., 2018). In the hippocampus of mice expressing human TAUP301L protein and AD patients with tauopathy, the TAU-injured neurons release the chemokine fractalkine CX3CL1 and an increase in the Nrf2 and HO-1 proteins levels (Lastres-Becker et al., 2014), suggesting an attempt of the diseased brain to limit microgliosis. The plasma and CSF levels of soluble CX3CL1 was reportedly found to be elevated in cognitive impairment and AD (Kim et al., 2008; Shi et al., 2011). The interconnection between CX3CL1 and NRF2 in the modulation of neuroinflammation was elucidated by Lastres-Becker et al. (2014) using the murine microglia (BV-2 microglial cell lines). The authors showed that CX3CL1 stimulation increases the NRF2-ARE gene expression by activating AKT (phospho-AKTSer473), leading to the inhibition of GSK3B by phosphorylation of Ser9 (phospho-GSK-3βSer9). On the contrary, by using primary microglia from mouse strains that lack either NRF2 (Nrf2^−/−^) or CX3CR1 (Cx3cr1^−/−^) *in vitro* stimulation with CX3CL1 failed to induce the HO-1 expression. Of note, both knockout mice showed microgliosis and astrogliosis in case of neuronal TAUP301L expression, suggesting the CXCL1/NRF2/HO-1-dependent mitigation of the pro-inflammatory phenotype. Moreover, inhibition of GSK3β correlated with a stability in the Nrf2 levels, indicating that inhibition of GSK3β prevents Nrf2 degradation bypassing the KEAP1. Indeed, GSK3β activity in the Neh6 domain of NRF2 creates a degradation domain that is then recognized by β-TrCP (Rada et al., 2011, 2012). Therefore, the lack of CX3CR1/PI3K/AKT signaling results in the non-phosphorylated active conformation of GSK3β which in turn leads to the Nrf2 downregulation *via* GSK3β/β-TrCP pathway. A summary of the regulatory mechanism of Nrf2 in AD is reported in Figure 2.

## Role of Nrf2 in Parkinson's Disease

**Figure 3:**
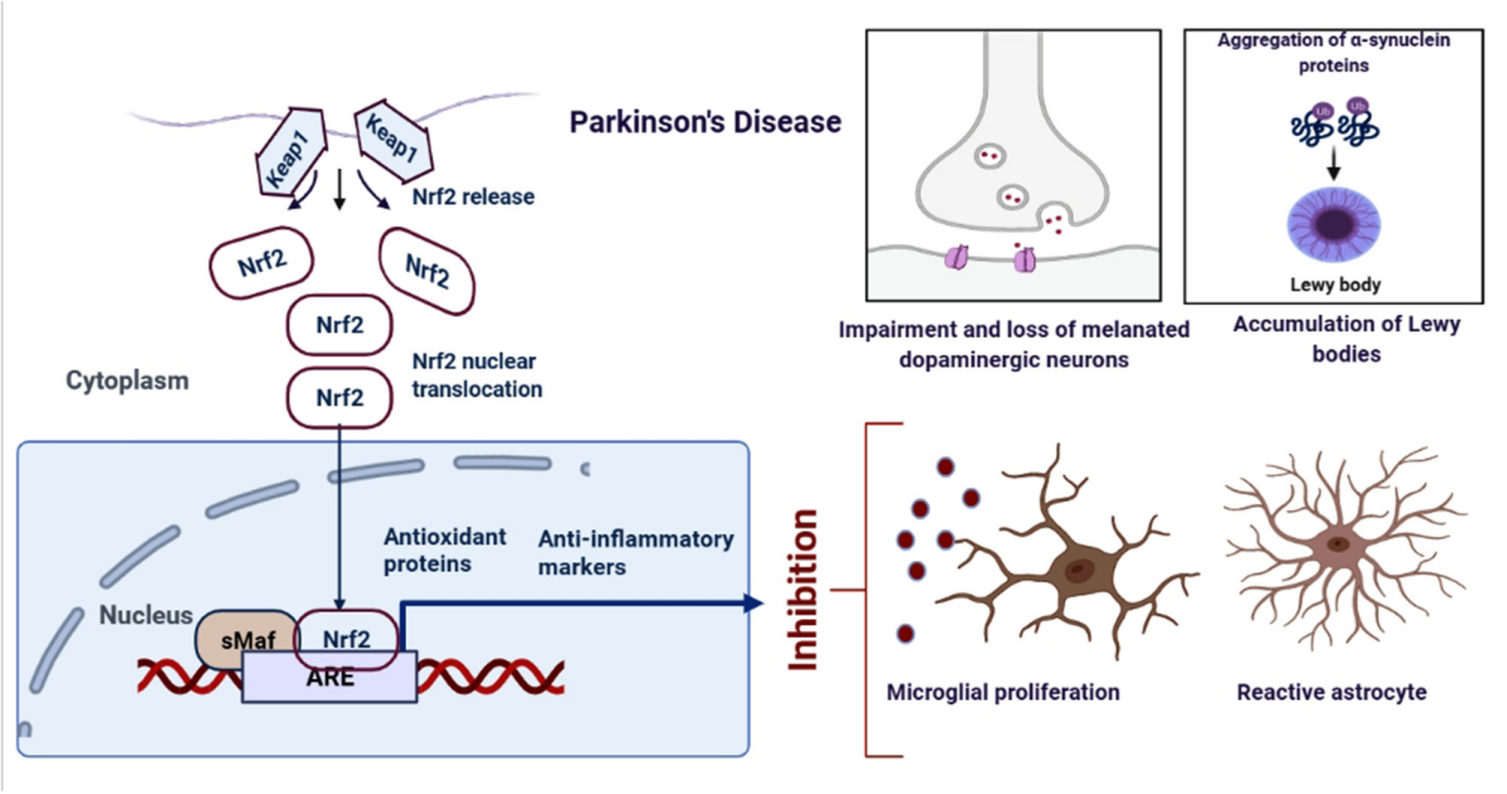
Schematic representation of the role of Nrf2 in PD. An upregulation in the dopamine release could result into the oxidative stress, increased ROS production and neuroinflammation. However, when astrocytes and microglia produces Nrf2, it activates the antioxidant and anti-inflammatory gene expressions.

Parkinson's Disease (PD) is another common neurodegenerative disorder affecting 1-4% of people above 65 (Miller and O'Callaghan, 2015). The pathological feature of PD includes the loss of dopaminergic neurons in the substantia nigra pars compacta and intraneuronal accumulations of a-synuclein into Lewy body inclusions (Figure 3). PD patients show resting tremor, postural instability, gait imbalance, bradykinesia, and dementia in some cases as the most characteristic symptoms (Vranová et al., 2014). Microglia, a cell type of the monocyte-macrophage linage, is mainly responsible for inflammation in the brain. PD is observed to be associated with the up-regulation of the free radical generating enzymes and the accumulation of CA-MO microglia (Pajares et al., 2020). The Nrf2 activity was found to be significantly reduced in the 1-methyl-4-phenyl-1,2,3,6-tetrahydropyridine (MPTP) model of PD, and loss of Nrf2 further exacerbated the phenotype (Chen et al., 2009). Mice and monkeys when treated with MPTP showed astroglial HO-1 expression in striatum and elevated iron deposition (Youdim et al., 2004). In support of these, several studies have demonstrated increased markers of oxidative damage along with decreased levels of antioxidants in the blood and CSF of PD patients, which was found to be linked with Nrf2 pathway (Dias et al., 2013; Wei et al., 2018).

Expression of Nrf2 was strongly nuclear in PD nigral neurons, whereas it is cytoplasmic in normal conditions (Ramsey et al., 2007). Furthermore, Nrf2 signature, represented by NQO1 and HO-1 expressions are up-regulated, suggesting Nrf2-dependent brain protection (van Muiswinkel et al., 2004; Cuadrado et al., 2009; Yoo et al., 2013). Another report showed the sequestering of NQO1 and p62 protein expressions in Lewy bodies in postmortem samples of PD patients, suggesting Nrf2-dependent neuroprotection (Lastres-Becker et al., 2016). Similarly, in a PD *Drosophila* model, Nrf2 overexpression and Keap1 knockdown attenuated the reduced locomotor activity and dopaminergic neuron degeneration (Nakabeppu et al., 2007; Barone et al., 2011).

Activated microglia play a key role in neuroinflammation by release of cytokines. Microglia are resident innate immune cells of the brain that act as macrophages, which ranges from pro-inflammatory M1 phenotype to immunosuppressive M2 phenotype. Activated microglia encompasses multiple functions: clearance of accumulated or deteriorated neuronal and tissue elements, dynamic interaction with neurons whilst regulating the synaptic pruning process, and maintaining overall brain homeostasis as well as maintenance of chronic inflammation (Moehle and West, 2015). A significant increase was observed in proinflammatory cytokines in the experimental models and cerebrospinal fluid of PD patients (Mogi et al., 1994, 1999; Blum-Degen et al., 1995; Starhof et al., 2018). *In vivo* findings confirmed that widespread microglial activation is associated with the pathological process in PD thus supporting the hypothesis that inflammation is a significant component of progressive dopaminergic degeneration *via* cytokine release (Cicchetti et al., 2002).

Evidence for a role of NRF2 in the modulation of microglial dynamics between pro-inflammatory M1 and anti-inflammatory M2 phenotypes was reported by Cuadrado's group (Rojo et al., 2010). In Nrf2-knockout mice with MPTP treatment, basal ganglia show a more severe dopaminergic dysfunction than wild type. Nrf2-deficient mice exhibited intense astrogliosis and microgliosis indicated by an increase in expressions of GFAP and F4/80, respectively. These changes were also associated with an increase in the levels of COX-2, iNOS, IL-6, and TNF-α with a significant decrease in anti-inflammatory markers such as FIZZ-1, YM-1, Arginase-1, and IL-4. These results were further confirmed in microglial cultures stimulated with apoptotic conditioned medium from MPP1-treated dopaminergic cells. These findings indicated a role of Nrf2 in tuning microglial activation in PD progression (Rojo et al., 2010).

Multiple studies investigated expression profiles at cellular levels and reports showed selective expression of Nrf2 in astrocytes as compared with neurons (Shih et al., 2003). Additionally, it has also been shown that astrocyte specific Nrf2 pathway activation confers protection to vulnerable neurons (Kraft et al., 2004).

Using a cell model derived from biopsies of the olfactory mucosa termed, human olfactory neurosphere-derived cells (hONS), it has been observed that a significant alteration of “Nrf2-dependent antioxidant response pathway” was associated to reduced levels of glutathione and salt, a measure of cellular metabolic activity based on reduction by NAD(P)H-dependent dehydrogenase enzymes in PD patient-derived hONS cell lines when compared with control-donor derived cells (Matigian et al., 2010). Another study, reported that knocking down Nrf2 with siRNA in control-donor derived hONS cells leads to the cellular phenotypes seen in PD cell lines (Cook et al., 2011). In this hONS model, Nrf2 pathway activation by treatment with L-Sulforaphane, restored disease-specific deficits in cellular functions (glutathione content and MTS metabolism) (Cook et al., 2011).

Reports showed the up-regulation in Nrf2 expression in the hippocampal cells of AD brain tissue (Lastres-Becker et al., 2014; Joshi et al., 2015; Liddell, 2017). DJ-1 (gene; PARK7) a recessively inherited Parkinson's gene, prevents oxidative stress in a Nrf2-dependent manner by preventing Keap1-mediated ubiquitination (Clements et al., 2006). In addition to this, a short, cell penetrating, peptide derivative of DJ-1 prevents H_2_O_2_, 6-OHDA, and DA-induced oxidative stress in Nrf2-dependent pathway in neuroblastoma cell lines (Lev et al., 2015). Furthermore, mutant isoforms, knockdown, and knockout models of DJ-1 have shown to prevent the expression of the Nrf2-mediated redox signaling molecules, thioredoxin 1 (Im et al., 2012) and glutathione (Zhou and Freed, 2005). Therefore, a strong relationship between Nrf2 and PD is well established till date and it has been proposed that intervening oxidative stress through the Nrf2-dependent pathway could be useful in the treatment of PD.

Another line of evidence revealed the key role of phosphatidylinositol 3-kinase (PI3K) and Akt kinases in the activation of the Nrf2-mediated antioxidant response (Martin et al., 2004; Salazar et al., 2006; Lim et al., 2008; Rojo et al., 2008). The regulation of neuroprotection in PD involving PI3K/Akt/GSK3β signaling axis is supported by the fact that Akt activity declines with age, which is the main risk factor for sporadic PD (Ikeyama et al., 2002). Additionally, an association between PD and single nucleotide polymorphism has been reported in the GSK3B gene leading to the elevation of GSK3β expression and activity (Kwok et al., 2005). Furthermore, inhibition of GSK3β enhanced Nrf2 activity and increased expression of Phase II antioxidant genes, thereby protecting against oxidants such as H_2_O_2_, 6-hydroxydopamine (6-OHDA) and MPP ^+^ (Salazar et al., 2006; Jain and Jaiswal, 2007; Rojo et al., 2008). A summary of the regulatory mechanism of Nrf2 in PD is reported in Figure 3.

## Cross-Talk Between Nrf2 and Neuroinflammation in AD and PD

It is assumed that Nrf2 and nuclear factor kappa-light-chain-enhancer of activated B cells (NF-κB) signaling pathways cooperate for the maintenance of the physiological tissue homeostasis and for the regulation of the cellular response to stress and inflammation. Several pro-inflammatory cytokines regulated by NF-κB, such as TNF-α, IL-1β, IL-6 and matrix metallopeptidase 9 (MMP9), have been shown to be enhanced in Nrf2-deficient mice in neuroinflammation as compared with wild-type mice, indicating that Nrf2 silencing promotes NF-κB -mediated inflammation (Mao et al., 2010).

Since a bidirectional connection occurs between the brain and the peripheral immune system, therefore, Nrf2 plays a crucial role in neuroinflammation (Jarrott and Williams, 2016). In neurodegenerative disorders, microglial cells become activated, leading to the release of pro-inflammatory cytokines and the production of ROS and reactive nitrogen species (RNS) (Hoenen et al., 2016). To circumvent the damaging effect of oxidative stress and inflammation, cells have developed several defense mechanisms. Nrf2/ARE signaling influences anti-inflammatory changes in many kinds of brain injuries, such as subarachnoid hemorrhage, traumatic brain injury, ischemia and neurodegenerative disease (Yan et al., 2008; Zhang et al., 2010; Wu et al., 2014). The Nrf2 activation pathway promotes the expression of antioxidative response elements, whereas nuclear factor kappa B (NF-κB), a protein complex which influences cytokine production and cell survival, also promotes cellular responses to neuronal injury and synaptic plasticity (Shih et al., 2015). Aging *per se* is associated with Nrf2 dysfunction and continuous, low-grade inflammation. These processes further contribute to neurodegeneration. So far, results have shown that neuroinflammation has a vital role in the progression and development of AD and PD and the imbalance between Nrf2 and Nuclear factor kappa B (NF-κB) could contribute to their pathogenesis of neuroinflammation.

Considerable evidence has revealed oxidative stress as well as chronic inflammation and autophagy in the brain correlated with the slow deterioration of AD (Prasad, 2017). In addition, experimental data so far suggest that all these changes are associated with impaired Nrf2 activity (Pajares et al., 2016; Zhang et al., 2019). The impaired spatial learning and memory abilities of mice and the accumulation of Aβ and p-tauS404 in the hippocampus were observed to be aggravated in a mouse model of AD (APP/PS1 transgenic (AT) mice with genetic removal of Nrf2 (Ren et al., 2020). Furthermore, astroglial and microglial activation was exacerbated along with upregulation of the proinflammatory cytokines IL-1β, IL-6, and TNF-α (Ren et al., 2020).

Since, inflammation is implicated as the key mechanism that actively contributes to neurodegeneration, by influencing the responses of microglia and astrocytes. Nrf2 is the key regulator for the two important cytoprotective pathways, anti-inflammation and anti-oxidation. Nrf2, which is essential for protection against oxidative/xenobiotic stresses, has been shown to block transcriptional upregulation of the proinflammatory cytokines IL-6 and IL-1β in myeloid cells by binding to the proximity of proinflammatory genes that guide the macrophage activation toward the M1 proinflammatory phenotype (Kobayashi et al., 2016). Treatment of neural stem/progenitor cells with Aβ induced reduction of neuronal differentiation which was prevented by Nrf2 over-expression, while Nrf2 deficiency enhanced impairment of neuronal differentiation (Kärkkäinen et al., 2014). In the same study, the Aβ40 treatment had no direct effect on neurosphere proliferation, however, when associated with Nrf2 overexpression it led to an enhanced proliferation of neurospheres and Nrf2 deficiency reduced neurospheres proliferation. Knockout of Nrf2 in mice crossed with AT or mutant HsAPPV717I/HsMAPTP301L mice showed exacerbated astrocyte and microglial activation (Lastres-Becker et al., 2014; Joshi et al., 2015). However, treatment with the kavalactone methysticin, an Nrf2 activator, significantly reduced microglial infiltration, astrogliosis, and the secretion of the proinflammatory cytokines TNF-α and IL-17A in APP/Psen1 mice (Rojo et al., 2010). Furthermore, Nrf2 deletion exacerbated the inflammatory response in AT mice as shown by increased proinflammatory factors and hippocampal astrogliosis and activation of microglia surrounding the Aβ plaque, and NFTs (neurofibrillary tangles) in AD animal models or human patients (Griffin et al., 1989; Simard and Rivest, 2006; Lok et al., 2013; Licht-Murava et al., 2016). Indeed, hyperphosphorylated tau protein and fibrillar Aβ lead to inflammatory processes and the release of IL-1β *in vivo* and *in vitro* (Halle et al., 2008; Sarlus and Heneka, 2017). Other reports showed that Aβ accumulation induced progressive impairment in microglial cells in their ability to phagocytize Aβ (Shi and Holtzman, 2018). However, Nrf2 absence induced more aggressive activation of astroglia and inflammation in AT mice, which might be through activation of the NF-κB pathway (Fragoulis et al., 2012; Buendia et al., 2016).

Microglia-mediated neuroinflammation is also a crucial pathological process and the key factor is glial activation, especially microglial activation. It has already been reported that chronic Nrf2 deficient microglia leads to neuroinflammation and AD, and loss of Nrf2 primed microglia toward inflammatory phenotype with an increase in Clec7a and CD68 markers (Yu et al., 2019). Nrf2 knockout in aged mice leads to increased reactive microglia, proinflammatory cytokines, and infiltrating immune cells in the brain with AD-like impairment. Nrf2 knockout microglia showed increased NF-κB p65 and CD86 suggesting inflammatory phenotypes. Moreover, loss of Nrf2 leads to the reduction in microglial expressions of P2ry12, Tmem119, Gpr34, Tgfbr1, and Mafb, suggesting the Nrf2-dependent regulation of microglial homeostasis.

On the other hand, astrocytes are also well-known for active involvement in AD disease progression by influencing accumulation of amyloid plaques, neuroinflammation, and oxidative stress. It has been shown that Presenilin 1 mutated (PSEN1ΔE9) AD patient astrocytes have altered cytokine secretion upon inflammatory stimulation and exploits higher oxidative metabolism, thereby leading to the high production of ROS than healthy control astrocytes (Oksanen et al., 2017). The same study shows that inflammation activates the metabolism of human astrocytes. In a consecutive study, it has been shown that Nrf2 activation reduces amyloid secretion, cytokine release, with a subsequent increase in GSH secretion in AD astrocytes (Oksanen et al., 2020). Activation of Nrf2 also enhances the metabolism of astrocytes and increases the utilization of glycolysis.

It is well established that inflammation is a complex interplay of different pathways. In this context, heme oxygenase-1 (HO-1, HMOX1, EC 1.14.99.3), is an inducible 32 kDa protein which is responsible for the catalysis of the rate-limiting step of oxidative heme degradation which converts heme into three bioactive products namely free iron, carbon monoxide (CO) and biliverdin. Biliverdin is further rapidly converted into bilirubin and plays crucial roles in inflammation, apoptosis and oxidative stress (Wunder and Potter, 2003). Nrf2 directly regulates the expression of the HMOX1 gene responsible for the activity of HO-1 enzyme. Several *in vitro* and *in vivo* experiments have shown the role of Nrf2-dependent HO-1 expression for the anti-inflammatory activity. In presence of AD, HO-1 is observed to be increased in the temporal cortex and hippocampus in human brains (SantaCruz et al., 2004). Hippocampal expression of mutated tau induces increase in HO-1 and GCLC transcripts in wild type mice but not in Nrf2 knockout mice, indicating the crucial role of Nrf2 in the reduction of oxidative stress and inflammation (SantaCruz et al., 2004).

Apart from this, NF-κB p65 subunit downregulated the Nrf2-ARE pathway at transcriptional level by competitive interaction with the CH1-KIX domain of CREB-binding protein (CBP), which results in Nrf2 inactivation, or by the recruitment of the corepressor histone deacetylase 3 to ARE, thus promoting local histone hypoacetylation (Liu et al., 2008). Accordingly, silencing of p65 by knockdown promotes Nrf2 complex formation with CBP (Liu et al., 2008).

A study in primary cultured astrocytes from Nrf2 wild type or knockout mice exposed with oxyhemoglobin (OxyHb) has shown activation of NF-κB and an up-regulation of downstream pro-inflammatory cytokines in astrocytes. Moreover, this up-regulation was much greater in knockout astrocytes than in wild type astrocytes (Pan et al., 2011). Recently a study was conducted in a transgenic mouse that combines amyloidopathy and tauopathy with either wild type (AT-Nrf2-WT) or Nrf2-deficiency (AT-Nrf2-KO) (Rojo et al., 2017, Rojo et al., 2018). The results showed that AT-Nrf2-WT mice died prematurely, at around 14 months of age, due to motor deficits and a terminal spinal deformity, whereas AT-Nrf2-KO mice died roughly 2 months earlier. Nrf2-deficiency mice showed exacerbated astrogliosis and microgliosis, with a significant increase in GFAP, IBA1 and CD11b levels. However, treatment with Nrf2 activator, dimethyl fumarate (DMF) showed a reduction in pro-inflammatory mediators COX2 and NOS2, as well as the gliosis markers GFAP, IBA1 and MHCII with a significant increase in the expression of Nrf2, Nqo1, Osgin1, and Gstm1 in the brain, thereby preventing cognition and motor complications (Rojo et al., 2017, 2018). With aging, the cerebral blood vessels of Nrf2-deficient mice showed enhanced senescence markers, aging-induced vascular inflammation and blood-brain barrier leakage (Fulop et al., 2018; Tarantini et al., 2018) along with white matter leukoencephalopathy (Hubbs et al., 2007).

Astrocytes provide trophic support for neurons, promote formation and function of synapses, and prune synapses by phagocytosis, in addition to fulfilling a range of other homeostatic maintenance functions (Sofroniew and Vinters, 2010). Astrocytes undergo a dramatic transformation called “reactive astrocytosis” after brain injury and disease. Liddelow and colleagues have shown that activated microglia is able to induce pro-inflammatory astrocytes, designated as A1-astrocytes, *via* secretion of IL-1α, TNFα, and C1q both *in vitro* and *in vivo*. This subset of astrocytes changes their expression profile and phenotype to form neurotoxic reactive astrocytes. Indeed, A1s lose the ability to promote neuronal survival, outgrowth, synaptogenesis and phagocytosis, and induce death of neurons and oligodendrocytes (Liddelow et al., 2017). The authors also show that A1s are highly present in human neurodegenerative diseases including AD and PD. Silencing by either knockout gene or antibody drugs for IL-1α, TNFα, and C1q inhibit A1 reactive astrocyte formation, therefore, this pathway has a therapeutic value in neurodegenerative diseases (Liddelow et al., 2017). A prolonged dysfunction of astrocytes and microglia activation reportedly accelerate the degeneration of SNpc dopaminergic neurons during early dysfunction induced by 6-OHDA lesion in rats (Kuter et al., 2018). Upon activation to the M1 phenotype, microglia secrete pro-inflammatory cytokines and neurotoxic molecules leading to the inflammation and cytotoxic responses. In contrast, the M2 polarized microglia secrete anti-inflammatory cytokines such as IL-4 and IL-10, neurotrophic factors (e.g., BDNF and IGF-1), and extracellular matrix proteins such as fibronectin (Subramaniam and Federoff, 2017).

With age, the failure of the astrocytic Nrf2-antioxidant axis response upon inflammation and oxidative stress significantly influences VM astrocyte-microglia-neuron interactions (Chinta et al., 2013; L'Episcopo et al., 2013; Silva-Palacios et al., 2018; Serapide et al., 2020). At the SNpc level, aging-induced decline of astrocytic Nrf2 gene expression promotes an up-regulation of major microglial proinflammatory gene expressions, such as *TNF-*α*, IL1*β*, IL-6* and *Nos2* both at striatal (Okamoto et al., 2011; L'Episcopo et al., 2013) and SNpc (L'Episcopo et al., 2011, 2018) levels, further accelerates oxidative stress and inflammation.

In a very recent study, NRF2 knockout and wild-type mice that overexpress human α-Syn (hα-Syn^+^/Nrf2^−/−^ and hα-Syn^+^/Nrf2^+/+^ respectively) were developed and an increased phosphorylation and oligomerization of α-Syn was observed in hα-Syn^+^/Nrf2^−/−^ mice. Further analysis showed a loss of tyrosine hydroxylase expressing dopaminergic neurons in the substantia nigra with amplified oxidative stress, higher inflammatory markers including COX-2 and iNOS-2 levels and an increased autophagic burden, especially in the midbrain, striatum and cortical brain regions (Anandhan et al., 2021).

## Potential Nrf2 Activators Toward Neuroinflammation Clinical Trials

So far, we discussed different aspects of Nrf2 signaling pathway in oxidative stress and inflammation in neurodegenerative diseases, therefore, it is also worthwhile to discuss compounds and natural products which could modulate Nrf2-dependent treatment of neuroinflammation. Extensive research has been focused till date on identifying the agents/factors that regulate the association between Nrf2 and Keap1 and there are many chemical compounds, and natural products that have been identified as Nrf2 activators in neuroinflammation (Cuadrado et al., 2019). In this context, dimethyl fumarate (DMF), is the only drug so far approved by US Food and Drug Administration and marketed by Biogen, as an anti-inflammatory therapeutic agent in multiple sclerosis with the ability to inhibit inflammation *via* Nrf2 antioxidant pathway (Linker et al., 2011; Gold et al., 2012). More importantly, in a pre-clinical study performed in an animal model of PD, it was observed that the oral administration of DMF protected nigral dopaminergic neurons against α-synuclein toxicity and reduced astrocytosis and microgliosis from stereotaxic delivery to the ventral midbrain of recombinant adeno-associated viral vector expressing human α-synuclein (Lastres-Becker et al., 2016). Furthermore, *in vitro* studies showed that the neuroprotective effect of DMF was associated with the altered regulation of autophagy markers SQTSM1/p62 and LC3 in MN9D, BV2 and IMA 2.1 and a switch in microglial phenotype toward a less pro-inflammatory type (Lastres-Becker et al., 2016). DMF and its bioactive metabolite monomethylfumarate (MMF) activate *in vitro* the Nrf2 pathway by promoting S-alkylation of Keap1 and by determining nuclear exit of the Nrf2 repressor Bach1 (Ahuja et al., 2016). Nrf2 activation by DMF was associated with glutathione depletion, decreased cell viability, and inhibition of mitochondrial oxygen consumption and glycolysis rates, whereas MMF determined an increase of these activities. However, despite these differences, both DMF and MMF showed neuroprotective effects and blocked neurotoxicity in a mouse model of PD. Interestingly, this effect was not observed in Nrf2 null mice (Ahuja et al., 2016). Mechanistically, Linker and colleagues demonstrated that in an animal model of chronic multiple sclerosis DMF treatment improved preservation of myelin, axons and neurons (Linker et al., 2011). In the same study, *in vitro* experiments demonstrated that fumarates were able to increase murine neuronal survival and to protect human or rodent astrocytes against oxidative stress. Moreover, it was observed that MMF was able to promote Nrf2 activation by determining a direct modification of Keap1 at cysteine residue 151 (Linker et al., 2011). More recently, a study performed in human astrocytes demonstrated that cytoprotective activity of MMF is mediated by the upregulation of the oxidative stress induced growth inhibitor 1 (OSGIN1)-61 kDa isoform (Brennan et al., 2017). MMF-induced OSGIN1 expression is NRF2-dependent and modulates inflammatory markers thus contributing to cell protection against oxidative challenge (Brennan et al., 2017).

More recently, Bach1 inhibitor by vTv Therapeutics (High Point NC, USA) proved to be effective against MPTP-induced dopinergic neurodegeneration *via* Nrf2 activation (Ahuja et al., 2016). Another compound has been identified to bind Keap1 in the *in vitro* assay in the low micromolar range, by Biogen Idec, Merrimack Pharmaceuticals, Celgene Corporation (USA), Evotec AG (Germany), and NoValix (France) (Marcotte et al., 2013). Another compound within the isothiocyanate group of organosulfur compounds, Sulforaphane (SFN), activates Nrf2 in the basal ganglia leading to the upregulation of phase II antioxidant enzymes HO-1 and NQO1 (Jazwa et al., 2011). Importantly, SFN treatment activates Nrf2-dependent pathway to restore glutathione and MTS metabolism in PD hONS cultures (Cook et al., 2011). In wild-type mice, SFN protected against parkinsonian toxin methyl-4-phenyl-1,2,3,6-tetrahydropyridine (MPTP)-induced death of nigral dopaminergic neurons by reducing astrogliosis, microgliosis, and release of pro-inflammatory cytokines (Jazwa et al., 2011). Similar effects have also been shown by SFN treatment in other animal models of PD (Trinh et al., 2008; Morroni et al., 2013; Advedissian et al., 2016). Sulforaphane, originally isolated from Brassicaceae plants, has been enrolled in clinical trials (NCT04213391) for the treatment of AD based on Nrf2 activation. However, sulforaphane is relatively unstable at room temperature. In this context, its synthetic analogs such as SFX-01 (Evgen Pharma developed drug) are attracting considerable attention for AD drug development.

The expression of HO-1 in microglial cells was observed to be responsible for the anti-inflammatory effect of compounds such as schizandrin C (Park et al., 2013) and several other compounds (Foresti et al., 2013). Moreover, in tauopathy, NRF2- and fractalkine receptor-knock out mice did not express HO-1 in microglia, suggesting their crucial role in the mitigation of neuroinflammation (Lastres-Becker et al., 2014). Cryptotanshinone, a monomer compound, can attenuate LPS-induced neuroinflammation *via* Nrf2/ HO-1 signaling pathway in BV-2 microglial cells (Zhou et al., 2019).

Quercetin, a natural flavonoid, significantly attenuated the LPS-induced synaptic loss in the cortex and hippocampus of the adult mouse brain (Khan et al., 2018). Quercetin protects against mitochondria dysfunction and progressive dopaminergic degeneration of neurons in experimental models of PD (Ay et al., 2017). Quercetin also shows an improvement in cognitive impairment in 6-OHDA-induced PD (Korczyn, 2001). In another study, quercetin prevents NO and iNOS over-expression in PC12 cells and down-regulates pro-inflammatory genes expressions (IL-1ß, COX-2 and TNF-α) in zebrafish (Zhang et al., 2011).

Treatment with synthetic triterpenoids such as CDDO-methyl amide (2-cyano-N-methyl-3,12-dioxooleana-1,9(11)-dien-28 amide; CDDO-MA) of neuroblastoma SH-SY5Y cells resulted in Nrf2 activation and translocation from cytosol to nucleus and significant protection against MPTP-induced nigrostriatal dopaminergic neurodegeneration, pathological α-synuclein accumulation and oxidative damage in mice (Yang et al., 2009). CDDO-MA treatment of fibroblasts from wild type, but not from Nrf2 knockout mice, inhibited ROS production induced by t-butylhydroperoxide by promoting the activation of ARE genes (Yang et al., 2009). The two structural analogs of CDDO (TP-319 and TP-500), obtained by the cyclization of squalene, have demonstrated improved blood-brain-barrier permeability, and protected against oxidative stress and inflammation in MPTP-induced dopaminergic neurotoxicity in mice (Kaidery et al., 2013). This activity was Nrf2-dependent as treatment of Nrf2 knockout mice with these CDDO analogs failed to inhibit MPTP neurotoxicity and to induce Nrf2-dependent cytoprotective genes (Kaidery et al., 2013). Another potent Nrf2 activator, KKPA4026 was identified by virtual screening of the Asinex and Chemdiv databases (Kim et al., 2020). KKPA4026 was demonstrated to induce the expression of the Nrf2-dependent antioxidant enzymes heme oxygenase-1, glutamate-cysteine ligase catalytic subunit, glutamate-cysteine ligase regulatory subunit, and NAD(P)H:quinone oxidoreductase 1 in BV-2 cells. Furthermore, in the MPTP-induced mouse model of PD, KKPA4026 was able to reduce behavioral deficits and protected dopaminergic neurons in an Nrf2-dependent manner. Similarly, a recent report showed a novel therapeutic candidate ALGERNON2 (altered generation of neurons 2) reduced the proinflammatory cytokines secretion and stabilized cyclinD1/p21 complex by inhibiting Dyrk1A activity, leading to Nrf2-dependent antioxidant and anti-inflammatory responses in a MPTP-induced PD model (Kobayashi et al., 2016). Interestingly, this compound enhanced neuronal survival also in other neuroinflammatory conditions, particularly in the transplantation of pluripotent stem cell–derived dopaminergic neurons into murine brains, thus confirming the therapeutic potential of ALGERNON2 in neuroinflammation-triggered neurodegeneration conditions (Kobayashi et al., 2016).

## Conclusions

Oxidative damage and neuroinflammation are the key regulators in the pathogenesis of AD and PD. Therefore, one way to prevent these oxidative stress and inflammation is to upregulate the endogenous protection system in the neuronal cells. Nrf2-Keap1 signaling pathway is the hallmark of redox signaling and controlled neuroinflammation. Here, we reviewed ongoing scientific literature about regulation of Nrf2 signaling pathway in different aspects of neuroinflammation and related cognitive impairment. However, there are still some mechanisms such as interactions between Nrf2 and JAK/STAT signaling in neuronal cells that needs to be studied. Although many clinical studies and pharmaceutical companies are currently targeting Keap1, the key regulator of Nrf2, it is still challenging to enhance the targeting of these compounds against neurodegenerative disorders due to the targeted Nrf2 dissociation from Keap1 and its persistence in the nucleus as well as the permeability through the blood brain barrier as well as their proper biotransformation.

Furthermore, new chemical entities which have entered clinical trials for AD and PD therapy should be analyzed for Nrf2 response to determine their advantages in neuroinflammation.

## Author Contributions

SS, BB, EP, PT, and LS contributed to conception and design of the study. SS wrote the first draft of the manuscript. All authors contributed to manuscript revision, read, and approved the submitted version.

## Conflict of Interest

The authors declare that the research was conducted in the absence of any commercial or financial relationships that could be construed as a potential conflict of interest.

## Publisher's Note

All claims expressed in this article are solely those of the authors and do not necessarily represent those of their affiliated organizations, or those of the publisher, the editors and the reviewers. Any product that may be evaluated in this article, or claim that may be made by its manufacturer, is not guaranteed or endorsed by the publisher.
